# Taking on the Corporate Determinants of Ill-health and Health Inequity: A Scoping Review of Actions to Address Excessive Corporate Power to Protect and Promote the Public’s Health

**DOI:** 10.34172/ijhpm.2023.7304

**Published:** 2023-09-09

**Authors:** Benjamin Wood, Jennifer Lacy-Nichols, Gary Sacks

**Affiliations:** ^1^Global Centre for Preventive Health and Nutrition (GLOBE), Institute for Health Transformation, School of Health and Social Development, Faculty of Health, Deakin University, Geelong, VIC, Australia; ^2^Centre for Health Policy, The University of Melbourne School of Population and Global Health, Melbourne, VIC, Australia

**Keywords:** Corporate Power, Commercial Determinants of Health, Corporate Determinants of Health, Countervailing Power, Economic Democracy

## Abstract

**Background:** In many sectors of the economy, for-profit business corporations hold excessive power relative to some governments and civil society. These power imbalances have been recognised as important contributors to many pressing and complex societal challenges, including unhealthy diets, climate change, and widening socio-economic inequalities, and thus pose a major barrier to efforts to improve public health and health equity. In this paper, we reviewed potential actions for addressing excessive corporate power.

**Methods:** We conducted a scoping review of diverse literature (using Scopus, Web of Science, HeinOnline, and EBSCO databases), along with expanded searches, to identify state and collective actions with the potential to address excessive corporate power. Actions were thematically classified into overarching strategic objectives, guided by Meagher’s ‘3Ds’ heuristic, which classifies actions to curb corporate power into three groups: dispersion, democratisation, and dissolution. Based on the actions identified, we proposed two additional strategic objectives: reform and democratise the global governance of corporations, and strengthen countervailing power structures.

**Results:** We identified 178 documents that collectively cover a broad range of actions to address excessive corporate power. In total, 18 interrelated strategies were identified, along with several examples in which aspects of these strategies have been implemented.

**Conclusion:** The proposed framework sheds light on how a diverse set of strategies and actions that seek to address excessive corporate power can work synergistically to change the regulatory context in which corporations operate, so that broader societal goals, including health and equity, are given much greater prominence and consideration vis-à-vis powerful corporate interests.

## Background

 The rise of the for-profit business corporation (hereafter corporation, unless otherwise specified) has been described as one of the most fundamental global transformations of the past three centuries.^1,2^ Corporations are entities that owe the legal basis for their mandate and powers to pursue private profits to a combination of state concessions granted upon incorporation (see Box 1).^3,4^ In general, it is the *combination* of these concessions that give corporations considerable financial, economic, and political advantages over non-corporate business forms (eg, sole proprietorships) and non-business corporate forms (eg, incorporated universities).^4^

**Box 1. Key Rights and Privileges Conferred to Business Corporations Upon Incorporation in Many Jurisdictions, Adapted From Multiple Sources^2,4-7^**

- Legal separation of ownership and control
- Limited liability for shareholders
- Joint-stock mechanism allowing for the accumulation of pools of capital from multiple parties
- Right to an unlimited lifespan
- Right to own shares in other business entities
- Right to pursue multiple lines of business
- Right to operate in multiple jurisdictions
- A range of political rights, including the right to challenge legislation

Dating back at least to the 16th century when European states began to attach a special set of rights and privileges to business entities in the pursuit of national, imperial or public interest objectives,^2,8^ corporations have greatly impacted on the health of many populations.^2,7,9^ Corporations have been lauded by many for contributing to economic prosperity and development, job creation, and meaningful technological progress.^11-13^ However, many concerns have been raised about the myriad ways by which many corporations negatively impact on population health and health inequity.^2,7,9,14,15^ In recent years, an increasing body of research on the influence of corporations and other commercial actors on health and equity has fallen under the banner of the *commercial determinants of health*, referring to the ‘*systems, practices, and pathways through which commercial actors drive health and equity*.’^16^

 Public health stakeholders have long sought to expose and hold powerful business actors, especially corporations, to account for harmful practices.^17^ Many approaches to address harmful business practices have focused on the products and practices of particular industries, especially health-harming commodity industries and those related to essential healthcare products or services. Since the harms of cigarette smoking were exposed in the 1940s and 1950s, for instance, public health stakeholders have led international efforts for stricter regulation of tobacco products and of various practices conducted by tobacco corporations.^1,18-22^ As another example, public health campaigns targeting pharmaceutical corporations that jeopardise efforts to make medicines (eg, antiretrovirals against the human immunodeficiency virus) and vaccines (eg, against SARS-CoV-2) accessible and affordable for all have made some inroads into improving health equity for various populations.^23,24^ Industry-specific approaches to holding powerful corporations to account are important, and, in many cases, have played a substantial role in improving health outcomes. Nevertheless, many of the most pressing public health challenges of our time, including climate breakdown and widening socio-economic inequalities, are driven and reinforced by large corporations in diverse sectors and contexts. It follows, then, that cross-sectoral actions that seek to address the root causes of these problems are needed.^16,25-27^

 The concept of ‘excessive corporate power’ offers a potentially crucial entry point for identifying and linking such cross-sectoral approaches to improving and protecting population health and health equity.^28-30^ Albeit a contested term, excessive corporate power can refer to the capacity of corporate actors to ‘interfere’ on an ‘arbitrary’ basis with the real or perceived choices of other actors or groups (eg, workers, consumers, citizens, other businesses, legislators, and researchers).^31^ This definition draws from both Lukes’ (1974, 2005) definition of power and Pettit’s (1997) definition of domination, with the latter author referring to ‘arbitrary’ interference as interfering for the purpose of self-interest (eg, profit maximisation) with minimal regard to the interests of others affected.^31-33^ Several public health scholars have described how excessive corporate power, conceptualised as above or in a similar way, can influence health. These conceptualisations often cover relatively direct or instrumental mechanisms of influence, including by subjecting workers to harmful working conditions or poor wages, or by shaping the preferences of disadvantaged individuals and social groups via aggressive and predatory marketing practices.^34,35^ The conceptualisations of excessive corporate power also cover more indirect mechanisms of influence, such as the way in which corporations shape markets, supply chains, the distribution of wealth and income, public policy, regulation, science, the mainstream media, and public opinion, thereby *structuring* the real or perceived choices that particular actors can make to the detriment of their health or the health of others.^28-30,34-37^ These dynamics take play within broader systems that, in recent decades, have become increasingly neoliberalised and financialised (albeit to varying degrees), characterised by a suite of policies, norms and governance arrangements that have accommodated, rather than confronted, corporate power.^38-43^

 A few notable exceptions notwithstanding,^7,27,39,44-48^ discussions and research on how to curb excessive corporate powerremain relatively underdeveloped in the public health literature. With this in mind, this paper aimed to identify a diverse range of actions with the potential to address excessive corporate power. The underlying premise for the review was that *any *action that addresses excessive corporate power, at least as conceptualised above, has the *potential* to positively influence population health and health equity.

## Methods

 Given the complex and interdisciplinary nature of the subject at hand, we chose to conduct a scoping review of diverse social science and legal literature. We describe our theoretical and organising framework below, along with the scoping review methods used.

###  Theoretical and Organising Framework

 We drew from Meagher’s ‘3Ds’ heuristic device to guide our framing of identified accounts and prescriptions on how to address excessive corporate power.^9,49^ This heuristic categorises actions to address excessive corporate power into three groups — *dispersion*, *democratisation*, and *dissolution*.^9,49^ *Dispersion* refers to the decentralisation and redistribution of concentrations of corporate wealth and power. Within this domain, Meagher focuses mostly on antitrust (competition law) measures designed to prevent future monopolies and break up existing monopolies. *Democratisation* refers to ensuring that corporate decision-makers take into account the interests of all actors subject to excessive corporate power within their control (eg, via diverse stakeholder representation on corporate boards). Lastly, *dissolution* refers to dissolving excessive corporate power that cannot be dispersed or democratised, largely through revoking corporate privileges granted upon incorporation.

 We chose Meagher’s ‘3Ds’ heuristic to be our organising framework as we felt it was broad enough in scope to inform the categorisation of a wide range of potential actions. Nevertheless, we maintained flexibility during our categorisation process by allowing for the development of new categories in cases where identified actions did not neatly fit within the three original groups (further details below). We also chose Meagher’s heuristic because we felt it appropriately recognises the complex relationship that typically exists between states and corporations. In particular, the heuristic does not assume that states and corporations are always in contest, that the general rise in corporate power seen in recent decades has come at the expense of state power, nor that the boundaries between states and corporations are always distinct. Rather, in accordance with the concession and political theories of the corporation,^3,4^ it assumes that corporations cannot exist without states, and that, in principle, states have the power to regulate corporations within their jurisdiction.

###  Scoping Review Methods

 Following the process set out by Arksey and O’Malley,^50^ we conducted a scoping review to identify actions that have the potential to address excessive corporate power (see Supplementary file 1 for search terms used). Searches were completed across four databases: Scopus, Web of Science, HeinOnline, and EBSCO (encompassing Medline Complete, Business Source Complete, EconLit, Environment Complete, Global Health, Legal Source, and Political Science Complete). Databases were searched in January 2022. Search results (n = 327 studies) were downloaded and imported into Endnote citation software where duplicates (n = 92) were removed. Backwards citation searching was undertaken to identify additional studies (see Figure). Following Godin and colleagues’ approach to systematically analyse grey literature,^51^ two advanced Google Scholar and Google searches were completed (limited to English language), with the first conducted in January and the second in February 2022. The first 100 results for each search were scanned. The websites of organisations and think tanks identified through the Google searches were also examined for relevant documents. Finally, the list of included documents was supplemented with: (*i*) the authors’ knowledge of relevant documents, including some published after the literature searches were completed; and (*ii*) expanded searches on illustrative examples found during the review process that warranted further information to inform analysis.

**Figure:**
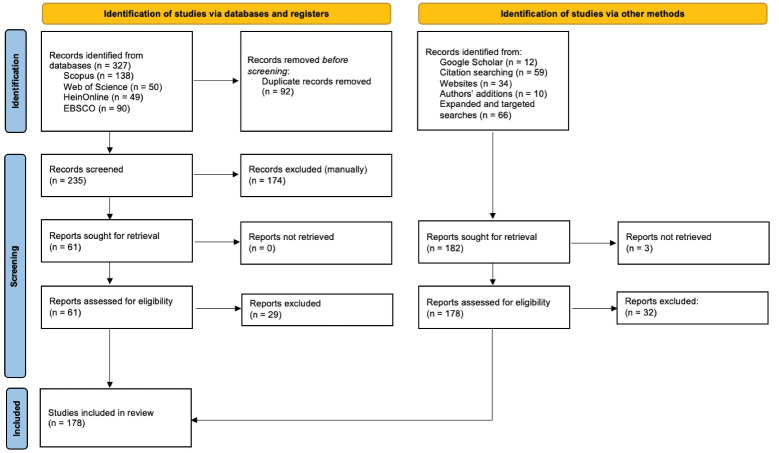
PRISMA Flow Diagram. Abbreviation: PRISMA, Preferred Reporting Items for Systematic Reviews and Meta-Analyses.

 No specific limits were placed on dates or geography, although documents not published in English were excluded. We screened the titles and abstracts (or table of contents and executive summaries where relevant) for all search results (see Table 1). Following screening, full texts were retrieved and tabulated by one of the authors in excel. From the documents that fulfilled the inclusion criteria, we extracted the title, authors, date of publication, the identified or prescribed action(s), and the corresponding country or region (where relevant).

**Table 1:** Inclusion and Exclusion Criteria

**Inclusion**	**Exclusion**
Published in English.	Not published in English (due to the language background and skills of the authors).
Provided an account of a realised action(s), or the prescription for a potential action(s), to address excessive corporate power. The author(s) needed to have problematised or conceptualised excessive corporate power in a manner consistent with our own conceptualisation described earlier. Actions could be state actions (eg, reforming or strengthening state instruments), private market-based actions (eg, consumer boycotts, shareholder activism), or any other collective action (eg, promotion of alternative business forms).	Did not include an account or prescription of ways to address excessive corporate power (problematised or conceptualised in a manner consistent with our own conceptualisation described earlier).

###  Data Analysis and Framework Development

 Identified actions were thematically grouped into strategies in an iterative manner. Guided by Meagher’s ‘*3Ds*’ heuristic,^9,49^ these strategies were grouped into overarching strategic objectives via a process of deductive coding. During the analysis, additional actions and strategies were identified that did not neatly fit within the heuristic. After discussion amongst all authors, two additional strategic objectives were developed to encompass these additional actions and strategies: ‘*strengthen countervailing power structures*’ and ‘*reform and democratise the global governance of corporations.’* We documented examples in which the identified actions had been implemented, including, in some cases, the key actors involved.

## Results

 We identified 178 documents that collectively describe a broad range of actions to address excessive corporate power. Table 2 outlines the five strategic objectives and 18 strategies identified in this study, as well as illustrative examples of actions. The strategic objectives, strategies, and actions discussed in this section are interlinked and, in general, complementary to one another.

**Table 2:** Strategic Objectives, Strategies, and Illustrative Examples of Actions to Curb Excessive Corporate Power, Adapted From Meagher^9,49^

**Strategic Objective**	**Strategy**	**Illustrative Example of Action**
Disperse concentrated corporate wealth and power	Strengthen antitrust regulation to protect and promote the welfare of all citizens	Widen objectives of antitrust policy to consider broader welfare concerns
	Limit corporate rent-seeking and cost externalisation	Legislate free access to essential medicines and healthcare services
	Redistribute concentrations of corporate wealth and income through progressive tax policy	Implement a robust tax on share repurchases
	Strengthen regulation of political contributions, corruption, and conflicts of interest	Regulate corporate contributions to political candidates and parties
Strengthen countervailing power structures	Strengthen the countervailing power of workers and consumers	Strengthen labour and unionisation laws
	Strengthen transparency mechanisms to promote corporate accountability	Implement public country-by-country reporting requirements for transnational corporations
	Promote socially responsible shareholding	Support divestment campaigns targeting harmful industries
	Support legal remediation for citizens harmed by corporations	Support the use of *qui tam* suits by citizens against corporations
	Organise alternative modes of business and systems of production and distribution	Scale-up alternative forms of enterprise, such as worker co-operatives and mutual enterprises
Democratise corporate decision-making	Improve stakeholder representation on corporate boards	Mandate stakeholder representation requirements on corporate boards
	Mandate the pursuit of stakeholder value	Amend the objectives of the corporation under the law
	Mandate corporate decision-makers to identify and mitigate adverse social and environmental impacts	Implement robust corporate due diligence laws that consider human rights and environmental sustainability
	Increase public takeover of privatised ‘public goods’	(Re)municipalise public goods and services, such as water, energy, housing, and transport
Reform and democratise the global governance of corporations	Reform and democratise existing international organisations and institutional arrangements that sustain corporate power	Assign a greater role to national parliaments in the negotiation and ratification of WTO agreements
	Develop new international organisations and institutional arrangements that constrain corporate power	Revive plans to develop global institutions to govern transnational corporations
Dissolve excessive and harmful corporate power	Dissolve excessive and harmful corporate power	Revoke the privileges granted via incorporation of corporations that repeatedly violate regulations and/or human rights
	Wind-down harmful industries	Scale-up industrial policy that drives systematic transition from non-renewable to renewable energy sources
	Reform/transform the corporate form	Revoke limited liability for all corporations above a certain size in terms of assets or revenue

Abbreviation: WTO, World Trade Organisation.

###  Disperse Concentrated Corporate Wealth and Power

####  Strengthen Antitrust Regulation to Protect and Promote the Welfare of All Citizens 

 In many jurisdictions, antitrust or competition law represents one of the most powerful levers a state has at its disposal to disperse excessive corporate power, including through addressing high market concentration and abuses of market dominance.^9,52-54^

 The US antitrust model has been one of the most influential globally, both due to US influence over other national antitrust models, as well as the global influence of US corporations subjected in some way to US antitrust law. With one of the longest antitrust traditions, the US passed its first federal antitrust law in 1890 to supposedly preserve open markets and economic opportunities, as well as to safeguard society and democracy against extreme concentrations of wealth and power.^52-54^ Many contend, however, that US antitrust regulation has weakened considerably since the 1970s and 1980s, largely underpinned by a shift in thinking, led by the Chicago School and sponsored by big business, about the normative purpose of antitrust policy.^9,55-57^ The Chicago School contended that the sole objective of antitrust should be to advance ‘consumer welfare,’ a concept they considered synonymous with ‘economic efficiency,’ and one that is often narrowly interpreted as low consumer prices.^9,56^ Many antitrust agencies and courts around the world today continue to recognise ‘consumer welfare’ in such narrow terms as one of the primary goals of antitrust policy.^58,59^

 Some jurisdictions, however, have wider antitrust policy objectives than those prescribed by *consumer welfarists*, or at least are in the process of widening such objectives, that are arguably better aligned with the strategic objective of dispersing corporate power.^60-63^ For instance, one of the stated objectives of the Republic of Korea’s Monopoly Regulation and Fair Trade Act is to prohibit the excessive concentration of economic power.^64^ South Africa, as another example, has a model in which its antitrust agencies and courts must consider a set of public interest considerations, some which relate to the social and economic welfare of its so-called ‘Historically Disadvantaged Persons,’ that was reportedly established to help restore a society deeply divided along racial and socio-economic lines.^65^ In 2021, South Africa’s antitrust regulators blocked a merger *solely* on the grounds that it would have drastically reduced the shares held by ‘Historically Disadvantaged Persons’ in the target company.^66^ Moreover, at the time of writing, the European Commission was in the process of considering the integration of environmental sustainability objectives into the antitrust policy of the European Union.^61^

 In the United States, President Biden signed an executive order in 2021 that provides the statutory basis for stronger whole-of-government approach to antitrust policy to better protect democratic accountability and the welfare of diverse groups across society.^67^ Perhaps even more indicative of Biden’s intent to challenge Chicago School-style antitrust regulation was his appointment of Lina Khan as chair of the Federal Trade Commission and Jonathan Kanter as head of the US Department of Justice’s Antitrust Division.^60^ Khan, a key figure of the anti-monopolist ‘New Brandeisian’ antitrust movement, pledged among other things to use the power of antitrust law to curb the power of ‘*Big Tech*’ in a dramatically new way.^68^ Under the leadership of Kanter, the US Department of Justice’s Antitrust Division reportedly litigated more mergers in 2022 than any fiscal year on record.^69^

####  Limit Corporate Rent-Seeking and Cost Externalisation

 An important means of dispersing corporate power is to limit corporate rent-seeking – the generation of income that is unearned (eg, through owning a scarce asset, such as a patent) or generated in an extractive manner (eg, by externalising costs onto society; by abusing a dominant market position). The generation of excessive profits by virtue of holding and misusing a position of market dominance, in principle, can be addressed via antitrust law (discussed in the previous section).^9^

 Cost externalisation is an important form of corporate rent-seeking, wherein corporations effectively generate rents by not being held financially accountable for the harms they cause.^9^ In many jurisdictions today, well-established regulations have been implemented to restrict how certain harmful products are made, packaged, and labelled, as well as marketed to specified population groups.^70,71^ Similarly, fiscal policy has also been recognised as an important tool to limit health-related cost externalisation, including through measures such as ‘sin taxes’ to reduce the population-level consumption of harmful commodities.^70,72,73^

 Fiscal policy can also play a key role in targeting and redistributing excessive corporate rents and externalised costs more broadly. Many people, for instance, have called for ‘windfall profit’ taxes targeting certain fossil fuel and COVID-19 vaccine manufacturing corporations in light of their recent record profits.^74,75^ As perhaps a more radical example, we identified a proposal to reform tax law to disincentivise all forms of aggressive advertising, including by ensuring that corporations cannot offset the cost of advertising against the profits they generate to reduce their taxable income.^76^

 International agreements and conventions have, in some cases, played an important role in facilitating the spread of national regulations targeting harmful commodity industries. In the 1970s, for example, public health and civil society actors drove the development of the International Code of Marketing of Breast-Milk Substitutes (the BMS Code), endorsed by the World Health Assembly in 1981, in an attempt to address the role played by the infant milk formula industry in undermining health and human rights.^77,78^ Notwithstanding its relatively poor implementation and enforcement, the BMS Code provides provisions targeting the harmful marketing of infant milk formula and similar products that states can incorporate into national laws.^34^ As an another example, after decades of internationalised efforts to strengthen regulation of the tobacco industry, the World Health Organization (WHO) Framework Convention on Tobacco Control (FCTC) entered in force as binding law in 2005 for all parties to the treaty.^79^ The FCTC has reportedly facilitated a drop in both tobacco smoking prevalence and exposure to second-hand tobacco smoke around the world.^79^ It has also contributed to the resolution of legal challenges put forward by the tobacco industry in favour of governments, including by providing a legal and evidential basis for such regulatory measures.^80^

 The exploitation of intellectual property rights has been described as a particularly harmful form of corporate rent-seeking.^81-83^ Such behaviour can be particularly problematic when corporations exploit their monopoly rights over particular technologies in a way that denies access to essential goods (eg, seeds, medicines, vaccines) on the basis of the ability to pay.^84,85^ Our review identified that some countries have challenged this form of exploitation. For instance, during the 1990s and early 2000s, Brazil and Thailand successfully pursued the goal of universal access to antiretroviral therapy against HIV/AIDS, in part through legislating free access to such treatment and scaling domestic capacity to produce generic medicines.^86^ At the international level, and largely though the organised efforts of public health, civil society and some state actors, World Trade Organisation (WTO) member states adopted the Doha Declaration on Trade-Related Aspects and Intellectual Property Rights (TRIPS) and Public Health in 2001, which provides national governments some agency to take measures to protect their public’s health within the relatively restrictive TRIPS framework.^24^

 Further examples of corporate rent-seeking identified in the literature include tax minimisation, tax evasion, and corporate welfare (ie, money or aid given to a corporation from a government).^87-89^ In this respect, important measures suggested include mandating that corporations apportion tax to countries according to the location of their assets, employment, and sales^90^; implementing a fair and adequate global minimum corporate tax rate^91^; strengthening tax collection and enforcement^92^; and strictly controlling which corporations receive tax incentives, exemptions, and subsidies.^88,92^ With respect to the last measure, 197 countries formally agreed to speed up efforts to eliminate ‘inefficient’ fossil fuel subsidies, which now exceed US$500 billion every year, at the 2021 United Nations (UN) Climate Change Conference.^93^

####  Redistribute Concentrations of Corporate Wealth and Income Through Progressive Tax Policy

 On top of redistributing corporate rents, tax policy has an important role in curbing excessive corporate power through restricting concentrations of excess wealth and income.^94,95^ Measures such as raising corporate statutory tax rates, or taxing a corporations’ stock, fall within this strategy.^94^ Relatedly, it has been argued that tax policy should encompass the regulation of the use of corporations by shareholders and company executives to maximise their own private wealth and income. In this respect, measures could include taxing share repurchases,^96^ strengthening capital income and gains tax policies,^92^ implementing or strengthening financial transaction taxes to disincentivise high-frequency trading,^92,97^ and penalising corporations that exceed a certain threshold for the ratio of payments their Chief Executive Officer receives relative to median employee pay.^98^

####  Strengthen Regulation of Political Contributions, Corruption, and Conflicts of Interest 

 It has been argued that, in many contexts, political contribution (including campaign finance) and anti-corruption reforms will be required to restrain corporate influence in policy-making and politics.^89,99-102^ Important measures identified during the review include bans, limits, and real-time disclosure of political contributions from corporations^103,104^; tightly regulating, through bans, waiting periods, and disclosure laws, the ‘revolving door’ between legislators and regulators and high-level positions in corporations^105^; and the implementation of mandatory lobby registers and ‘ministers’ diaries’ that require detailed real-time disclosures for corporate engagement with public officials.^106,107^ In the United States, many commentators have specifically called for an overruling of the 2010 *Citizens United v. Federal Election Commission* decision, which permits unlimited election spending by corporations using treasury funds.^108,109^

 Public health actors have called on governments, academic institutions, the media, and civil society to strictly regulate their interaction with corporations, especially those active in health-harming commodity industries, to better manage conflicts of interest.^46,110^ The FCTC is often portrayed as an exemplar instrument in this respect. FCTC’s Article 5.3 establishes rules at the international level to ban engagement between public health officials and the vested interests of the tobacco industry.^46,70^ Article 5.3 also calls for the protection of public health policies from the vested interests of the tobacco industry, stating that: ‘*Parties shall act to protect [public health] policies from commercial and other vested interests of the tobacco industry in accordance with national law*.’^46^ Calls have been made for similar treaties to be applied to other health-harming industries, such as alcohol and ultra-processed foods.^110^ One proposal takes this further by calling for a broader international convention on the *commercial determinants of health* that would focus on coordinating policy responses to a range of commercial practices, political processes, and related norms.^46^

 Efforts made by the Australian Government to manage conflicts of interest during the ongoing review of the Australian Dietary Guidelines, including a dedicated governance committee and a commitment to publishing a summary of meetings, correspondence, and relevant phone calls from external stakeholders during the review process, provides an example of innovation in this area at the national level.^111^

###  Strengthen Countervailing Power Structures

####  Strengthen the Countervailing Power of Workers and Consumers 

 In line with arguments put forward by North American economist John Galbraith,^112^ the power of workers and consumers can help to countervail excessive corporate power. Workers can exercise countervailing power, for instance, by joining unions, taking part in strikes and sit-ins, and by whistle-blowing. We identified several examples of governments supporting the countervailing power of workers, including by strengthening labour and unionisations laws, protecting the right to strike and to collectively bargain, and ensuring adequate protections for whistle-blowers.^113-119^

 Consumers can exercise countervailing power in various forms, including by joining consumer movements, as well as by taking part in consumer boycotts. A well-known case of this in action was the consumer boycott of Nestlé — triggered by exposés of the company’s undermining of child and maternal rights in disadvantaged parts of the world – which contributed to multiple forms of meaningful change, including the development of the WHO BMS Code described earlier.^78^ Robust consumer laws, including the strict regulation of aggressive and predatory marketing practices, and privacy laws (especially with respect to ‘Big Tech’) are recognised as important ways by which governments can support consumers.^70,78,100,120,121^

####  Promote Socially Responsible Shareholding

 Shareholders have access to certain rights and entitlements that can be leveraged to influence corporate decision-making. ‘*Shareholder activism*,’ where individuals or organisations acquire corporate shares and thus the right to participate in voting on particular corporate policies and strategies, represents a market-based action with the potential to influence corporate decision-making in the public interest.^70,122,123^ As an example, in response to shareholder pressure led by the non-governmental organisation ShareAction, Unilever announced commitment in 2022 to set a new benchmark for public reporting with respect to the healthfulness of its food products.^124^ In some cases, divestment campaigns have called on shareholders to divest from corporations active in harmful industries (eg, fossil fuels, tobacco, and certain weapons), as well as those active or based in controversial regions (eg, Israel, Russia, and Myanmar).^125,126^

 As governments and government agencies at different levels are often corporate shareholders, they can support these shareholder activism campaigns and divestment initiatives. In 2021, for instance, Boston Mayor’s Michelle Wu prohibited the use of public funds within her jurisdiction to invest in corporations that derive more than 15% revenue from fossil fuel, tobacco, and private prison operations.^127^

####  Strengthen Transparency Mechanisms to Promote Corporate Accountability 

 Strong corporate transparency mechanisms, among other things, can provide civil society and state actors with the necessary evidence to trigger or reinforce efforts (eg, litigation, consumer boycotts, legislative reforms) to hold dominant corporations to account.^46,92,128^

 Increasing corporate tax transparency is recognised as one particularly important component in ensuring corporate accountability, especially given the non-transparent and often secretive nature of corporate tax minimisation and avoidance.^129^ In this respect, public country-by-country reporting has been proposed as a tool to better monitor and address the tax minimisation and avoidance-related activities of transnational corporations, such as transfer pricing.^129^ More broadly, mandatory corporate disclosure on social and environmental issues has been described as an important means for civil society and governments to hold corporations to account.^46,128,130,131^ The Corporate Sustainability Reporting Directive recently proposed by the European Commission serves as a notable example of what would be a mandatory reporting framework that, in principle, aims to broaden what large corporations must disclose with respect to the implementation of their environmental, social, and governance policies.^130^

####  Support Legal Remediation for Citizens Harmed by Corporations 

 Supporting and promoting access to justice for citizens harmed by corporations can act as an important countervailing power structure for citizens insofar as it can help to redress harmful and exploitative practices that enable some corporations to generate profits and consolidate power.

 Litigation in particular has been widely used to curb the production of harmful commodities, recover externalised costs, and hold corporations to account for the harms they have caused to individuals or groups of individuals.^70^ Especially since the 1990s, it has been noted that litigation of the tobacco industry in the United States has been somewhat successful, at least relative to earlier periods. One particularly momentous settlement took place in 1998, in which four of the largest tobacco corporations were required to, inter alia, stop engaging in marketing practices that target children, and pay an annual compensation to the states for health-care related costs associated with tobacco smoking.^132^ In recent years, the fossil fuel industry has been increasingly targeted by lawsuits.^133^ It was even noted in a 2022 Intergovernmental Panel on Climate Change report that climate-change related litigation had become one of several important new avenues for shaping climate and environmental policy worldwide.^134^

 Compared to private lawsuits, *qui tam* lawsuits, referring to suits in which private individuals or organisations assist a prosecution on behalf of the government, have been described as having greater potential to prompt broader regulatory changes relating to dominant corporations.^135^ It was suggested that, in order to support and scale up this hybrid private-public enforcement approach, states could define a wider range of regulatory laws in which *qui tam* lawsuits could be used to seek compensation related to particular harmful corporate actions, such as those in violation of public health, human rights, and environmental laws.^135^

####  Organise Alternative Modes of Business and Systems of Production and Distribution

 An important strategy to challenge excessive corporate power is to promote and organise alternative modes of business that allow communities to bypass systems of production and distribution dominated by corporations (especially those primarily concerned with the short-term interests of their shareholders).^136-139^ Important examples include co-operatives and mutual enterprises, which are collectively owned by various actors such as consumers or workers, and are often driven by principles including mutual aid, equity, solidarity, and community development.^46^ It has been noted that business co-operatives managed and owned by workers, such as Mondragon in the Basque region of Spain and Cooperation Jackson in the U.S, can provide meaningful living wage jobs and foster community development.^140,141^ Renewable energy co-operatives, which are playing a key role in renewable energy transition in a number of European countries, have also been described as important enablers of community development.^142^

 We identified several examples of communities around the world redesigning local modes of food production and distribution in their quest for ‘*food sovereignty*,’ often under the leadership of the 200-million strong *Via Campesina* movement.^139,143,144^ Notably, the worldwide expansion of programs and policies instituting ‘*food sovereignty’* highlights the political salience of reconfiguring local food systems to benefit the livelihoods, health, and food security of citizens and communities.^144^ As an illustration, local government policy-makers in the Brazilian city of Belo Horizonte, a city considered to be a pioneer in addressing food insecurity, have implemented a set of integrated local-level policies and programs that seek to promote access to safe, quality, and nutritious food.^145^

 National governments can play an important role in supporting business alternatives to shareholder-oriented corporations, including via scaling-up social enterprises through sufficient public investment initiatives, progressive public procurement policies, and implementing supportive legal frameworks.^46^ In 2006, as a notable example, South Korea’s parliament introduced its Social Enterprise Promotion Act, which, among other things, reportedly inspired the Ministry of Agriculture, Food and Rural Affairs to introduce and support a business scheme designed to support rural communities.^146^ Similarly, in 2014, France’s parliament implemented a ‘Social and Solidarity Economy’ law to better support social enterprises in promoting and achieving sustainable local development.^147^

###  Democratise Corporate Decision-Making 

####  Improve Stakeholder Representation on Corporate Boards

 To democratise corporate decision-making, many scholars have advocated for mandating stakeholder representation on corporate boards in order to allow stakeholders subject to corporate power a say in how such power is exercised and distributed.^148-150^ This idea partly builds on existing corporate law models of co-determination, such as in Germany, wherein workers of large companies have the legal right to elect representatives to almost half of all supervisory board positions.^151,152^ Corporate law has also been used to achieve gender parity on corporate boards in Norway.^153^ More broadly, a number of scholars have suggested that a potential way to improve the representation of the general public in corporate decision-making could be to mandate the inclusion of public representatives on the boards of large corporations.^154^

####  Mandate the Pursuit of Stakeholder Value 

 Voluntary ‘*stakeholder value*’ corporate models, in which corporate decision-makers voluntarily commit to take into account the interests of a broad range of their stakeholders, have recently become available as a legal form in several jurisdictions.^9,155-157^ However, despite the emergence of these newer corporate forms, many argue that the prevailing view of corporate purpose in many contexts continues to be that publicly listed corporations should extract and distribute value for the primary benefit of their shareholders.^4,9,92,158,159^

 The considerable shortcomings of voluntary corporate pledges that claim to address escalating social and ecological crises have led to mounting calls for states to obligate corporate directors to internalise the interests of *all* stakeholders in their decision-making.^148,149,159,160^ Some scholars, for instance, have called for corporate purpose to be redefined under law,^9,159,161^ with an example text as follows: ‘[to create] *sustainable value within the planetary boundaries while respecting the interests of its investors and other involved parties*.’^159^ Similarly, corporate charters, which detail the rights and obligations of corporations,^4^ have been described as an instrument that could be operationalised by states to ensure corporations pursue stakeholder value.^100,104,162^ In the United States, pertinent examples of federal chartering proposals that would require large corporations to pursue stakeholder value are included in the Nader Group Report of 1976,^163^ Elizabeth Warren’s proposed Accountable Capitalism Act,^164^ and Bernie Sander’s Corporate Accountability and Democracy Plan.^165^

####  Mandate Corporate Decision-Makers to Identify and Mitigate Adverse Social and Environmental Impacts 

 The review identified several studies in which it was noted that corporate due diligence laws have the potential to regulate the corporate pursuit of stakeholder value through requiring corporate directors to identify and mitigate actual and potential adverse social and environmental impacts related to their decisions.^130,166-171^ A number of countries, such as Germany and France, have implemented corporate due diligence laws with respect to human rights, with France also expanding such laws to encompass environmental harms.^130,172^ In a recent development, the European Commission set out a proposal in early 2022 for a new directive on corporate *sustainability* due diligence, which would, in principle, legally hold directors of large European Union-based corporations to account for the adverse human rights, climate change and environmental consequences of their decisions.^130^

 Many scholars have argued that an important way to safeguard the rights and interests of citizens around the world from corporate violations is to subject transnational corporations to a legally binding international instrument on human rights.^168-171,173-177^ Drafted in 2003, the ‘*Norms on the Responsibilities of Transnational Corporations and Other Business Enterprises with Regards to Human Rights*’ (the Norms) provides an example of such an instrument, although this was ultimately rejected by the UN Commission on Human Rights.^166,178^

 One innovative proposal identified during the review largely based on the principle of ‘restorative justice’ involved requiring all corporations above a certain size to prepare and continuously improve a ‘justice plan,’ referring to a plan developed via a deliberative process between corporate decision-makers and stakeholders to determine what must be done to prevent and repair injustices caused by the corporation.^179^ Under this proposal, large corporations would be required to improve their justice plans each year, and to monitor whether the citizens they affect are receiving just treatment.^179^

####  Increase the Public Takeover of Privatised “Public Goods”

 Many scholars have called for the ownership and control of privatised and outsourced ‘public good’ industries (eg, public utilities such as water) to be retransferred to communities or the relevant level of government through the processes of remunicipalisation, renationalisation, and rebuilding public sector capacities.^89,180-183^ A recent report from the Transnational Institute provides more than 800 examples of the remunicipalisation of public services, including water, energy, housing, transport, security, finance, and school canteens, in 1600 cities and 45 countries.^184^ A well-documented case occurred in 2000 in Bolivia, where, in response to large-scale and coordinated protests, the Bolivian government reversed the privatisation of Cochabamba’s water supply and handed back control to the city.^185^ As part of the ‘*energy democracy’* movement, as another illustration, an increasing number of cities across the world are calling for, and in some cases achieving, a transition towards the public ownership of their energy utilities.^186,187^

 The potential benefits of public control of technology, including the ways in which socially meaningful technologies and their associated benefits are distributed, are also well described.^81^ While not necessarily publicly owned, Cuba’s state-owned biotechnology and pharmaceutical sector has been lauded for successfully supporting its national health system, as well as fostering technology transfer among low- and middle-income countries (LMICs), providing somewhat of a contrast to the highly financialised biotechnology and pharmaceutical sectors in countries like the United States.^81,188,189^

 It was argued that governments and universities have an important role to play in promoting the public ownership of science, including as a means of protecting the processes of generating, disseminating, and using evidence to inform public policy from being captured by powerful corporate interests.^46,104^ In this respect, suggested measures included increasing government support for publicly-funded research (especially critical social science research), as well as strengthening the management of conflicts of interests that invariably arise from corporate-sponsored research (as alluded to in an earlier section).^46,104^

###  Reform and Democratise the Global Governance of Corporations

####  Reform and Democratise Existing International Organisations and Institutional Arrangements That Sustain Corporate Power

 We identified a number of proposals relating to reforming existing international organisations and arrangements to shift decision-making power from powerful states and their corporations back to elected governments and civil societies.^1,169,190-193^ Chimni, for example, proposes a suite of measures, including assigning a greater role to national parliaments in the negotiation and ratification of WTO agreements to ensure that the consent of the representatives of citizens in the respective country, where relevant, is adequately sought.^190^ To initiate such a measure, it was suggested that national parliaments could pass a law requiring consultation and consent as a precondition for ratification of any significant international agreement, including but not limited to those related to trade and investment.^190^ In its report entitled ‘A Fair Globalization,’ the International Labour Organisation called for greater flexibility to be given to countries for entering or opting out of proposed disciplines or issues in the WTO, including by allowing greater policy space for countries to pursue diverse national policy objectives.^191^ The Doha Declaration introduced earlier shows that some concessions, albeit limited, have been made under the auspices of WTO to protect national policy space in certain areas relating to public health (eg, access to medicines).

 The Investor State Dispute Settlement (ISDS) mechanism has come under considerable criticism for readily allowing transnational corporations to sue governments for implementing public health regulations.^194,195^ Several LMICs — including Argentina, Bolivia, Venezuela, Ecuador, South Africa, and Indonesia — have taken action on this issue, including by withdrawing from trade and investment agreements that have facilitated ISDS.^194,195^ Under the auspices of the UN Commission on International Trade Law, discussions on reforming the system of ISDS are currently under negotiation amid calls by some experts for its abolition.^196,197^

 With respect to the International Monetary Fund (IMF) and the World Bank, the International Trade Union Confederation recently published reports calling for these organisations to stop their structural reform programs that promote economic policies and processes, such as liberalisation and privatisation, that often sustain the power of corporations headquartered in wealthy countries.^198,199^ Among other things, International Trade Union Confederation made the case that the current conditionality policies of the IMF should be changed to better align with priority sustainable development goals.^198^

####  Develop New International Organisations and Institutional Arrangements to Constrain Corporate Power

 Our review identified numerous proposals for the development of new international organisations and institutional arrangements to constrain the power of transnational corporations. As a pertinent example, it was noted that during the 1970s a collective project of many countries, referred to as the New International Economic Order (NIEO), started to call for, among other things, the development of new international institutions to govern transnational corporations.^200,201^ The NIEO project produced some mixed results, at least initially. In 1974, not long after then-Chilean President Salvador Allende called for the international community to address the ‘*economic power, political influence and corrupting action*’ of corporations, the UN Centre on Transnational Corporations was formed.^202^ Largely due to US and corporate opposition, however, the UN Centre on Transnational Corporations failed to build consensus for a legally binding Code of Conduct for Transnational Corporations, and was eventually abolished in 1992.^202^ In early 2023, nearly 50 years from when the project first emerged, delegates from over 25 countries met in Havana, Cuba, in an attempt to revive discussions about a NIEO in the UN General Assembly.^201^

 Perhaps the most comprehensive proposal to democratise the global governance of corporations identified during the review entailed the development of a Second Assembly of the UN directly elected by the citizens of the world.^203^ This Second Assembly, it was suggested, could be given the authority to organise international committees of democratically elected representatives to oversee the work of international organisations (eg, the WTO, the IMF, and the World Bank).^1^ The committees could be mandated to hear complaints made against these international organisations by citizen groups, and have the authority to take cases to the International Court of Justice or the International Criminal Court as required.^1^

###  Dissolve Excessive and Harmful Corporate Power

####  Dissolve Harmful Corporations 

 Corporations that consistently breach the public interest can be disempowered through the revocation of some or all privileges granted via incorporation.^49,101,182^ While such an idea may appear radical in many contemporary contexts, it is worth noting that corporations were regularly dissolved in the United States and Europe prior to the 20^th^ century.^9^ More recently, several companies in the United Kingdom were dissolved for fraudulently claiming COVID-19 pandemic related business support.^204^ The recent dissolution of Purdue Pharma, a corporation that fuelled the US opioid epidemic, also offers a glimpse of this approach in action.^205^

####  Wind-Down Harmful Industries

 When breaches of the public interest apply more broadly to an industry, some argue that measures to ‘wind down’ the industry in question should be considered.^206,207^ Through innovative industrial policy, public investment strategies, and corporate law, corporations active in industries in direct conflict with public and planetary health (eg, fossil fuels, tobacco, certain pesticides) could, in principle, be forced to be redesigned so that their operations specific to the industry in question are reduced, substituted, and, when necessary, prohibited.^208,209^ As an illustration, many states, including the United Kingdom, Canada, and France, have recently made public pledges to phase out the use of coal as a source of energy.^210^ As another example, New Zealand recently moved to ban the sale of cigarettes to people born after the year 2010.^211^ At the local and municipal levels of government, an increasing number of political leaders are reportedly taking part in planning a prompt and systematic transition from energy dependence on fossil fuels towards clean and renewable energy sources within their jurisdictions.^88,212^

 International coordination, however, is required to prevent harmful industries that have been wound down in one context from investing more heavily in other contexts with weaker regulatory arrangements. The global elimination of leaded petroleum in 2021 shows that sufficient international coordination can be achieved to ‘wind down’ an industry at the global level.^213^

####  Reform/transform the Corporate Form

 Many historical and contemporary commentators have called for stricter control and, in some cases, systematic revocation of the legal, political, and economic privileges of corporations. In the United States, for instance, hundreds of local and municipal governments have in recent times issued ordinances supporting the revocation of ‘corporate personhood’ (ie, the legal notion that a corporation is an entity separate from the people associated with it).^108^ Corporate chartering reforms have also been proposed that challenge the right of perpetual existence, including by requiring corporations to apply for the renewal of their privileges granted upon incorporation at the completion of a defined period (eg, ten years).^100,214^

 A number of scholars have argued for a systematic rethink of limited liability, contending that it promotes corporate irresponsibility and the externalisation of costs as a core profit-maximising strategy.^215,216^ To address this issue, measures such as revoking limited liability for large corporations and implementing a system of ‘equity’ fines (wherein offending corporations would be required to issue shares that would be controlled by a compensation fund) have been suggested.^182,215,217-219^ Relatedly, some scholars have recognised the need to strengthen corporate law to ensure that parent corporations hiding behind a corporate ‘veil’ — that is, controlling a subsidiary by being a major shareholder, and thereby being protected under limited liability — can still be held liable for gross misconduct.^220,221^ The case of James Hardie, a transnational corporation that manufactured and distributed the majority of asbestos in Australia, provides one illustration of how some corporations seek to avoid liability through complex restructuring. In this case, though, James Hardie was challenged by the state of New South Wales and the High Court of Australia, with the corporation subsequently required to establish and fund a charitable trust to cover relevant claims.^221^

## Discussion

###  Overview

 This study identified a wide range of implemented and proposed actions, across multiple levels of governance (eg, subnational, national, and international) and regulatory domains, that have the potential to challenge excessive corporate power. We categorised these actions into 18 strategies and five strategic objectives to provide insight into how they might be able to work synergistically. Notwithstanding the preponderance of literature originating from high-income countries, many of the identified actions have been implemented in diverse contexts. However, we recognise that most actions have not been widely adopted, and, in many contexts, there are considerable political, institutional, and cultural barriers to their implementation.

 Many of the strategies largely fall under the purview of democratically elected governments. This is particularly the case for the strategies largely contingent on law (eg, those related to antitrust regulation, corporate purpose and form, and tax), as well as government policy and intergovernmental relations (eg, reforming and democratising the global governance architecture). While government action is required for many strategies, there are nevertheless opportunities for other actors to support and reinforce these government actions. Such opportunities are perhaps most explicit for the strategies that fall under the strategic objective of ‘strengthen[ing] countervailing power structures.’ Workers, for instance, can take part in organised labour actions^118^; shareholders can use their privileged positions ‘within’ the corporation to shape corporate policy and strategy in the public’s interest^124^; and citizens, non-corporate business actors and communities can contribute to the scaling up of alternative forms of business.^140,141,144^ Furthermore, while it was beyond the scope of this paper to provide explanations of the factors that led to the implementation of identified actions, civil society actors (eg, health organisations, labour unions, consumer organisations, grassroots movements, activist academics, citizens and citizen groups) likely played important roles in driving many of the identified actions — both state and collective. Civil society actors can present a considerable countervailing force vis-à-vis excessive corporate power, such as by exposing and raising awareness of harmful corporate practices, advocating for government policy and law reforms, and challenging ideas and norms that sustain corporate power.^1,46,70,114^ As part of mobilised civil society efforts, actors in the field of public health have often played a key role in challenging excessive corporate power, at least in some contexts.^24,46,70^ Testament to this is the implementation of many national public health regulations against harmful corporate products and practices, the successful litigation against health-harming industries, and the development of numerous public health-oriented international frameworks and conventions (eg, FCTC).

 Many of the identified actions and proposals represent a reversal or response to neoliberal policies and programs (eg, remunicipalisation and other public ownership initiatives to counter privatisation), the contemporary neoliberal-style international economic order (eg, challenging the ISDS mechanism to protect national sovereignty), and shifts in corporate governance towards ‘maximising shareholder value’ (eg, laws mandating corporate-decision makers to consider and balance a broad range of interests). The effects of these policies, governance arrangements and norms vary considerably around the world, which perhaps partly explains why state and collective efforts to address excessive corporate power are piecemeal and highly diverse. Several other proposals instead seek to fundamentally reconfigure the relationship between business corporations and capitalist society — a relationship that has evolved substantially in recent centuries.^1^ In some cases these proposals draw from historical laws and regulatory frameworks, such as some of the prescriptions relating to corporate law (eg, changes to the privileges that corporations received upon incorporation). In other cases, including the proposal to develop a Second Assembly of the UN directly elected by the citizens of the world, they represent radical ideas yet to be enacted.

 The proposed framework supports and links existing work in the public health literature that outlines and seeks to develop integrated approaches to the *commercial determinants* of ill-health and health inequity.^26,46,48,70,104,158,222,223^ Similar to Wiist and Freudenberg, for instance, the proposed framework engages with a set of government levers, such as those related to corporate and antitrust laws, not often discussed in the public health literature despite their *potential* to protect and promote population health and health equity.^47,158^ In line with the recent Lancet series on the *commercial determinants of health,* the framework also considers the role of cross-sectoral national policies and regulations, international frameworks and conventions, and the scaling up of alternative business models to systematically reduce the social and environmental harms caused by powerful commercial actors.^46^

 There are several opportunities for public health researchers to take part in supporting the implementation of this paper’s prescribed agenda. Public health researchers, for instance, could seek to collaborate with key actors (eg, researchers, government actors, business actors) from diverse regulatory domains and geographical contexts to explore the political, cultural, and institutional feasibility of implementing some of the identified actions and proposed strategies. Such work could be facilitated by intergovernmental organisations such as WHO and the UN Conference on Trade and Development, both of which have experience in supporting and coordinating government responses to particular issues pertaining to excessive corporate power. As noted by Friel et al, WHO’s new focus on addressing the commercial determinants of ill-health and health inequity could help to promote greater and more cohesive action on these determinants.^46^ Among other benefits, this type of work could help public health researchers and advocates develop key competencies and sensitivities to better understand and engage with a range of technical and epistemic communities. It might also provide an opportunity for public health researchers to voice and infuse public health ideas and objectives into policy discussions in which they might not otherwise be explicitly considered. Nevertheless, it is important to recognise that some of the identified actions might be feasible and culturally appropriate in one context, but not another. Further research is needed to understand the context-specific opportunities and barriers for implementing the proposed strategies and actions identified in this review.

 In recognition of the key role that civil societies play in driving social and political change, a related opportunity for public health researchers to contribute to efforts to address excessive corporate power could be to support and engage with a broad range of civil society actors. For example, future public health advocacy could seek to increase engagement with diverse actors — such as representatives of movements related to climate change, degrowth, feminism, anti-monopoly, food and energy sovereignty, feminism, indigenous rights, and tax and debt justice, as well as consumer organisations and trade unions — to identify common goals and potential ways to pool capacities and resources to achieve these shared goals in different contexts. As we have argued in this paper, the goal of curbing excessive corporate power to promote health and equity could serve as an entry point to identify common objectives among such civil society actors, as well as a potentially powerful way to frame advocacy campaigns pushing for government intervention. These advocacy efforts could help to generate enabling environments for governments to act on some of the strategies proposed in this review.

###  Strengths and Limitations

 A strength of this paper is that it includes a review of a diverse range of literature, and integrated findings and discussions from multiple fields and disciplines. The normative and theoretical basis of the paper, and its organising framework, also drew upon well-established theories of the corporation.^3,4^

 This paper has several limitations. First, given the broad-ranging nature of the topic, the search terms used in the scoping review were relatively narrow, and, as such, the review would not have identified all the actions that have previously been identified as having the potential to address excessive corporate power. This is particularly likely to be the case for actions taken in many LMICs, given that the data we extracted were skewed towards high-income countries.

 Second, we acknowledge that the process of grouping and classifying actions identified during the review would have been influenced by the concepts of the theoretical framework adopted, as well as our own perspectives. We did use an iterative approach to allow for some flexibility in categorising identified actions that we felt did not neatly fit under the organising framework. Nevertheless, the use of a different organising framework may have led to the identification of different strategies and strategic objectives.

 Third, it was beyond the scope of this paper to identify and examine how each action has translated or could potentially translate into positive health and equity outcomes. The way in which particular actions may be implemented in different contexts and how their impact may vary based on local factors was not considered. These represent important avenues for future examination.

 Furthermore, the focus of this study was on the impact of for-profit business corporations on health, because it is this particular business form that has emerged to dominate economies and politics around much of the world.^1,2^ We recognise, though, that non-corporate businesses can also negatively influence health and equity.^224^ We also recognise that for many corporations, such as certain state-owned corporations, political objectives of the corporation and/or the state in which it is headquartered may trump the objective of generating profits. In such instances, especially if the state in question is not a representative democracy, some of the proposed strategies would become largely inapplicable.

 Lastly, it was beyond the scope of this paper to examine the factors and processes that have led to, or could facilitate, the adoption of the identified actions in diverse contexts. We recognise that many of these actions have not been widely adopted, and that, in many contexts, there are likely to be substantial barriers to their implementation. For instance, many state, corporate and private actors benefit from excessive corporate power, and are likely to take steps to sustain and protect such power.^40,42^ Relatedly, some have argued that efforts to reform many state instruments, including some of those discussed in this paper, in ways that give increased prominence to broader social and environment objectives will likely fail because these instruments are primarily designed to serve the interests of large corporations and their major beneficiaries.^136,225^ Nevertheless, this paper highlights a range of complementary ‘top-down’ and ‘bottom-up’ actions that have been implemented in practice. The proposed framework can guide future research and advocacy efforts as part of a solutions-oriented *commercial determinants of health *agenda.

## Conclusion

 The proposed framework presented in this paper consists of a range of measures that can change the regulatory context in which corporations operate so that broader societal goals, including health and equity, are given much greater prominence and consideration vis-à-vis powerful corporate interests. As such, the framework provides guidance for those seeking to identify upstream and integrated solutions to many pressing and complex societal challenges, including unhealthy diets, climate breakdown, and widening socio-economic inequalities. Many of the identified actions and proposed strategies require direct involvement from democratically elected governments, but such involvement will only likely come about through strong civil society advocacy and collective action.

## Acknowledgements

 We are very grateful to Michelle Meagher for some very helpful comments on an earlier draft of this paper.

## Ethical issues

 Not applicable.

## Competing interests

 Authors declare that they have no competing interests.

## Supplementary files

Supplementary file 1. Search Terms Used.
