# Supplementary material for: Taking on the Corporate Determinants of Ill-health and Health Inequity: A Scoping Review of Actions to Address Excessive Corporate Power to Protect and Promote the Public’s Health
Source: Int J Health Policy Manag. 2023 Sep 9;12:7304. doi: 10.34172/ijhpm.2023.7304 (PMC10590241; doi:10.34172/ijhpm.2023.7304)
Supplement: Supplementary file 1 — Search Terms Used. [file ijhpm-12-7304-s001.pdf]

Article title: Taking on the Corporate Determinants of Ill-health and Health Inequity: A Scoping Review of Actions to Address Excessive Corporate Power to Protect and Promote the Public's Health

Journal name: International Journal of Health Policy and Management (IJHPM)

Authors' information: Benjamin Wood[1]*, Jennifer Lacy-Nichols[2], Gary Sacks[1]

[1]Global Centre for Preventive Health and Nutrition (GLOBE), Institute for Health Transformation, School of Health and Social Development, Faculty of Health, Deakin University, Geelong, VIC, Australia.

[2]Centre for Health Policy, The University of Melbourne School of Population and Global Health, Melbourne, VIC, Australia.

***Correspondence to**: Benjamin Wood, Email: b.wood@deakin.edu.au

Citation: Wood B, Jennifer Lacy-Nichols J, Sacks G. Taking on the corporate determinants of ill-health and health inequity: a scoping review of actions to address excessive corporate power to protect and promote the public's health. Int J Health Policy Manag. 2023;12:7304. doi:10.34172/ijhpm.2023.7304

Supplementary file 1. Search Terms Used

Category groups (and synonyms):

#	Concept	Search terms
1	Corporate power	"corporate power"
2	Actions	action* OR polic* OR law* OR legislat* OR intervention
3	Counter/challenge	address* OR curb* OR counter* OR challeng* OR check*
